# Metabolomics Reveals Metabolic Characteristics of Functional Cure in Chronic Hepatitis B Treated With Entecavir Combined With Pegylated Interferon Alpha

**DOI:** 10.1155/bmri/1031453

**Published:** 2026-09-03

**Authors:** Junling Wang, Xianghong Kong, Huiping Xu, Wei Zhang, Peng Peng

**Affiliations:** ^1^ Department of Hepatology, Heze Mudan People′s Hospital, Heze, Shandong, China; ^2^ Department of Infectious Diseases, Heze Mudan People′s Hospital, Heze, Shandong, China; ^3^ Department of Medical Imaging, Heze Mudan People′s Hospital, Heze, Shandong, China; ^4^ Department of Laboratory Medicine, Heze Mudan People′s Hospital, Heze, Shandong, China

**Keywords:** chronic hepatitis B, entecavir, functional cure, metabolomics, pegylated interferon alpha

## Abstract

**Background:**

Entecavir (ETV) combined with pegylated interferon alpha (PEG‐IFN*α*) improves chronic hepatitis B (CHB) functional cure rates, but therapeutic heterogeneity and underlying metabolic mechanisms remain unclear. This study used untargeted metabolomics to identify metabolic signatures, mechanisms, and predictive biomarkers of functional cure with ETV‐PEG‐IFN*α*.

**Methods:**

Thirty‐eight CHB patients were grouped into ETV monotherapy (Group E, *n* = 12) and ETV‐PEG‐IFN*α* combination therapy (Group Z, *n* = 26); Group Z was subdivided into cured (Group A, *n* = 13) and noncured (Group B, *n* = 13). Serum metabolomic profiling, multivariate statistics, and Kyoto Encyclopedia of Genes and Genomes (KEGG) pathway analysis identified differential metabolites. A random forest model was built using key metabolites.

**Results:**

Three hundred eighty‐eight metabolites were identified. Four differential metabolites distinguished Group A and B (upregulated guanidinoacetic acid, uracil 5‐carboxylate; downregulated L‐methionine S‐oxide, oleamide), enriching amino acid metabolism pathways. Nine differential metabolites between Group E and Z implicated amino acid, immune, and fatty acid pathways. The random forest model based on the four Group A/B metabolites showed 88.5% cross‐validation accuracy (AUC = 0.920), with L‐methionine S‐oxide and oleamide as key predictors.

**Conclusions:**

This study reveals metabolic rewiring in CHB functional cure via ETV‐PEG‐IFN*α* therapy, involving energy metabolism, oxidative stress, and immunomodulation, based on which we propose a tentative metabolism‐immunity synergy model to guide future research. Key metabolites, especially L‐methionine S‐oxide and oleamide, show exploratory predictive potential for functional cure that warrants further validation in independent cohorts.

## 1. Introduction

Chronic hepatitis B (CHB) remains a major global health challenge, with approximately 254 million individuals living with hepatitis B virus (HBV) worldwide and 1.1 million annual HBV‐related deaths—predominantly due to liver cirrhosis and hepatocellular carcinoma (HCC) [1, 2]. Functional cure, defined as hepatitis B surface antigen (HBsAg) seroclearance with sustained hepatitis B virus deoxyribonucleic acid (HBV DNA) negativity, represents the ideal therapeutic endpoint in current CHB management, as it significantly reduces the risk of disease progression. However, monotherapy with either nucleos(t)ide analogs (NAs) or immune modulators such as pegylated interferon alpha (PEG‐IFN*α*) achieves HBsAg clearance rates below 5% [3, 4]. A meta‐analysis demonstrated that compared with monotherapy, combination therapy with NAs and PEG‐IFN*α* significantly improves the HBsAg seroclearance rate [5]. Among combinatorial regimens, entecavir (ETV) plus PEG‐IFN*α* demonstrates superior efficacy [6, 7], yet substantial interpatient heterogeneity persists—where certain individuals attain functional cure while others exhibit suboptimal responses. To date, research on the metabolic regulatory mechanisms underlying this heterogeneous response remains scarce. Understanding these mechanisms is crucial for elucidating the mode of action of combination therapy and providing a theoretical basis for optimizing treatment strategies. Metabolomics, as an emerging systems biology approach, holds exceptional promise for unraveling host‐virus interplay and treatment responses. This technique enables comprehensive profiling of low‐molecular‐weight metabolites, providing a direct readout of physiological and pathophysiological states [8]. Through metabolomic investigations, biomarkers and metabolic pathways linked to disease progression and therapeutic efficacy can be identified [9]. This study is aimed at characterizing the metabolic reprogramming induced by ETV‐PEG‐IFN*α* combination therapy in CHB patients. By analyzing metabolites in CHB patients after treatment, we seek to understand how combination therapy alters the metabolic network in patients and the relationship between these alterations and therapeutic outcomes. Additionally, we will screen for metabolic markers associated with functional cure. By comparing metabolic profiles between patients who achieved functional cure and those who did not, we aim to identify key metabolites that could serve as potential biomarkers for predicting treatment responses. Furthermore, based on our findings, we developed a “metabolism‐immunity” synergy model. This model deserves further exploration, as it could inform future strategies, enhance CHB management, and contribute to achieving functional cure.

## 2. Materials and Methods

### 2.1. Study Subjects

We retrospectively accessed and screened on January 1, 2024, a total of 38 eligible patients with CHB who visited our hospital between June 2, 2021, and December 30, 2023. Based on their treatment regimens, they were divided into the ETV monotherapy group (ETVZ, hereinafter referred to as Group E) (*n* = 12) and the ETV‐PEG‐IFN*α* combination therapy group (ABZH, hereinafter referred to as Group Z) (*n* = 26). Group Z was further divided into CHB functional cure group (AZYZ, hereinafter referred to as Group A) (*n* = 13) and noncured group (BWYZ, hereinafter referred to as Group B) (*n* = 13) based on treatment outcomes. Following routine testing, all serum samples were collected and stored in a −80°C freezer until metabolomics analysis was conducted for this study from March 14 to May 20, 2024. To protect patient privacy, researchers had no access to any information that could identify individual participants during the data collection and statistical analysis stages. All samples were processed using anonymized codes. This study strictly adhered to the ethical guidelines of the Declaration of Helsinki and was approved by the Institutional Ethics Committee (Approval No.: LL2025003). Written informed consent was obtained from all participants.

### 2.2. Inclusion Criteria

All participants must meet the following requirements: (a) aged 18–60 years; (b) diagnosed with CHB according to the “Update on Prevention, Diagnosis, and Treatment of CHB: AASLD 2018 Hepatitis B Guidance” [10].

Group Z required: (a) received ETV treatment for at least 2 years; (b) baseline HBsAg ≤ 1500 IU/mL, hepatitis B e antigen (HBeAg) negative, and HBV DNA < 100 IU/mL; (c) willing to receive interferon therapy and signed informed consent. Group E required: (a) baseline HBV DNA < 100 IU/mL, HBeAg‐negative status, and HBsAg > 1500 IU/mL; (b) maintained HBsAg > 1500 IU/mL at 1‐/3‐month follow‐ups post 48‐week ETV therapy.

### 2.3. Exclusion Criteria

Group Z: (a) interferon allergy; (b) decompensated cirrhosis (current/history); (c) severe organic diseases (cardiac/pulmonary/renal/cerebrovascular/retinopathy); (d) comorbidities (autoimmune/psychiatric disorders, diabetes, thyroid dysfunction); (e) confirmed/suspected HCC or malignancies; (f) organ transplantation (prior/planned); (g) current immunosuppressant use; (h) pregnancy/lactation/planned pregnancy within 2 years; (i) substance addiction; (j) coinfection with human immunodeficiency virus (HIV), hepatitis C virus (HCV), hepatitis A virus (HAV), hepatitis E virus (HEV), or other liver diseases. Group E: same as items b–j of Group Z exclusion criteria.

### 2.4. Treatment Regimens

Patients with CHB in Group Z received 48 weeks of treatment with ETV (0.5 mg/day) combined with PEG‐IFN*α*‐2b (180 *μ*g/week), followed by a 24‐week follow‐up period during which PEG‐IFN*α*‐2b was discontinued and ETV monotherapy (0.5 mg/day) was maintained. Those who maintained HBsAg < 0.05 IU/mL and HBV DNA < 10 IU/mL at the end of follow‐up were defined as the cured subgroup; those with HBsAg > 0.05 IU/mL or HBV DNA >10 IU/mL after completing the same treatment regimen were defined as the noncured subgroup. Patients in Group E continued oral treatment with ETV (0.5 mg/day) throughout the study period.

### 2.5. Sample Collection and Processing

Following treatment completion, 4 mL of fasting venous blood was collected from CHB patients. All blood samples were allowed to clot at room temperature for 20 min, then centrifuged at 4°C and 3000 g for 10 min to separate serum. The serum was subsequently aliquoted into Eppendorf (EP) tubes and stored at −80°C for subsequent metabolomic analysis.

### 2.6. Clinical Laboratory Testing

Liver and kidney function indices were detected using an automatic biochemical analyzer (Model AU5821, Beckman, United States), including alanine aminotransferase (ALT), aspartate aminotransferase (AST), albumin (Alb), *γ*‐glutamyl transferase (GGT), alkaline phosphatase (ALP), total bilirubin (TBil), blood urea nitrogen (BUN), and creatinine (Cr). Complete blood count was analyzed using an automatic hematology analyzer (Model BC‐5100, Mindray, China). Quantitative detection of HBsAg, HBeAg, hepatitis B surface antibody (HBsAb), hepatitis B e antibody (HBeAb), and hepatitis B core antibody (HBcAb) was performed using an automatic chemiluminescence analyzer (Model Wan200 +, Youmike, Xiamen). All quantitative detections were conducted by magnetic particle chemiluminescence immunoassay using kits produced by Wantai Kerui (China), with a sensitivity of 0.05 IU/mL for HBsAg. HBV DNA in CHB patients was quantitatively detected using a real‐time quantitative PCR analyzer (Model ABI 7500, Thermo Fisher, United States) before enrollment. After treatment completion, HBV DNA was detected using the COBAS AmpliPrep/COBAS TaqMan automatic nucleic acid purification and fluorescent PCR analysis system, with a lower limit of detection of 10 IU/mL.

### 2.7. Untargeted Metabolomics Analysis

#### 2.7.1. Reagents and Instruments

Liquid chromatography‐mass spectrometry (LC‐MS) grade methanol (MeOH) was purchased from Fisher Scientific (Loughborough, United Kingdom). 2‐Amino‐3‐(2‐chlorophenyl)‐propionic acid was obtained from Aladdin Reagents (Shanghai, China). LC‐MS grade acetonitrile (ACN) was purchased from Fisher Scientific (Loughborough, United Kingdom). Formic acid was obtained from TCI (Shanghai, China). Ammonium formate was purchased from Sigma–Aldrich (Shanghai, China). Ultrapure water was prepared using a Milli‐Q water purification system (Millipore, Bedford, United States). Ultra‐performance liquid chromatography (UPLC) analysis was performed using a Thermo Vanquish system (Thermo Fisher Scientific, United States), and mass spectrometry analysis was conducted using a Thermo Orbitrap Exploris 120 mass spectrometer (Thermo Fisher Scientific, United States).

#### 2.7.2. Sample Preparation [11] and Analysis

Experimental samples were thawed at 4°C and vortexed for 1 min to ensure thorough mixing. An appropriate volume of each sample was then accurately transferred to a 2 mL centrifuge tube, followed by the addition of 400 *μ*L methanol and vortex mixing for 1 min. The mixture was centrifuged at 12,000 rpm for 10 min at 4°C, and the entire supernatant was transferred to a new centrifuge tube for concentration and drying. Finally, the samples were reconstituted with 150 *μ*L of a solution containing 80% methanol‐water and 2‐chloro‐L‐phenylalanine (4 ppm). After filtration through a 0.22 *μ*m filter membrane, the filtrate was injected into a detection vial for LC‐MS analysis. Detailed conditions for UPLC/mass spectrometry (UPLC‐MS) analysis of samples are provided in Supplementary Material 1.

#### 2.7.3. Data Processing and Analysis

To monitor system stability and data quality, quality control (QC) samples were prepared by pooling equal volumes from all study samples and injected at regular intervals throughout the analytical run. Principal component analysis (PCA) of QC samples was performed to assess analytical reproducibility (Figure S1), and the relative standard deviation (RSD) of metabolite intensities across QC samples was calculated. Metabolites with RSD > 30% in QC samples were excluded from further analysis to ensure data reliability (Figure S2).

First, raw mass spectrometry files were converted to mzXML format using the MSConvert tool in the ProteoWizard software package (V3.0.8789) [12]. The R XCMS (V3.12.0) package was used for peak detection, peak filtering, and peak alignment [13] to generate a metabolite quantification list. Subsequently, support vector regression correction based on QC samples was performed to eliminate systematic errors. Metabolite identification (database searching) was conducted using public databases including HMDB [14], MassBank [15], LipidMaps [16], mzCloud [17], KEGG [18], and the self‐built standard library of PANOMIX (Suzhou PANOMIX Biomedical Tech Co. Ltd), with the parameter set as mass deviation < 30 ppm, yielding qualitative results of metabolites.

Dimensionality reduction analyses, including PCA, partial least squares‐discriminant analysis (PLS‐DA), and orthogonal partial least squares‐discriminant analysis (OPLS‐DA), were performed on sample data using the R package ropls [19]. Permutation tests with 200 random permutations were subsequently performed to evaluate the risk of model overfitting; a Q^2^ intercept < 0.05 after permutation was defined as the criterion to exclude overfitting. For QC of metabolomic profiling, metabolites with RSD > 30% across all QC pooled samples were eliminated prior to downstream multivariate modeling, and QC‐based support vector regression correction was implemented to remove systematic instrumental deviation, ensuring the reliability of metabolite quantification even with the limited sample size of 38 participants. All permutation test statistics and QC quantitative metrics were archived in Table S1 and Figures S3A,B. The impact and explanatory power of each metabolite′s content on sample classification were evaluated based on *p*‐values from statistical tests, variable importance in projection (VIP) values from OPLS‐DA, and fold change (FC) between groups, to assist in screening marker metabolites. Metabolites with *p*‐values (or false discovery rate, FDR) < 0.05 and VIP values > 1 were considered to have statistically significant differences. Functional pathway enrichment and topological analyses of the screened differential metabolites were performed using the MetaboAnalyst [20] package. The enriched pathways were visualized to locate differential metabolites in metabolic pathways using the KEGG Mapper tool.

### 2.8. Statistical Analysis

All data were statistically analyzed using SPSS 28.0 (IBM, United States) and R 4.3.1 (rstatix package). Quantitative data with normal distribution and homogeneous variance were expressed as mean ± standard deviation (mean ± SD). Comparisons between groups were performed using one‐way analysis of variance (one‐way ANOVA), and pairwise post‐hoc comparisons were conducted using the Bonferroni method. Nonnormal or heteroscedastic data were presented as median (interquartile range) and analyzed using the Kruskal–Wallis H test, with post‐hoc comparisons via the Dunn test. Categorical variables were expressed as frequencies and analyzed using Fisher′s exact test. A *p*‐value < 0.05 was considered statistically significant.

To address the significant baseline difference in HBsAg between Group E and Group Z (*p* < 0.001), three complementary approaches were applied: (1) analysis of covariance (ANCOVA) on 388 log‐transformed metabolites, adjusting for baseline HBsAg as a continuous covariate with Benjamini–Hochberg FDR correction; (2) confounder‐corrected OPLS‐DA using HBsAg‐adjusted residuals, validated by 7‐fold cross‐validation and 1000 permutation tests; and (3) partial correlation analyses controlling for HBsAg to verify independence of metabolite‐group associations.

The random forest algorithm [21] was employed to construct a predictive model comprising 500 decision trees. Differential metabolites identified between Group A and B served as input features, which were standardized using Z‐score normalization. To optimize hyperparameters and mitigate overfitting risk, a nested 5‐fold cross‐validation strategy was implemented. Model performance was evaluated by the area under the receiver operating characteristic (AUC) curve, with 95% confidence intervals calculated through 1000 bootstrap resampling iterations. All analyses were conducted in Python 3.10 environment using scikit‐learn (V1.4.0).

## 3. Results

### 3.1. Baseline Characteristics of Study Population

As presented in Table 1, Groups A and B demonstrated comparable baseline characteristics, with no statistically significant differences in gender distribution, median age, baseline HBsAg levels, liver function parameters, or hematological indices, confirming sufficient comparability between the two subgroups receiving combination therapy. Group E exhibited significantly higher baseline HBsAg levels compared with Groups A and B, consistent with its inclusion criterion of HBsAg > 1500 IU/mL. Importantly, all other clinical parameters showed no significant differences between Group E and the combination therapy groups, except for the predetermined HBsAg threshold.

**Table 1:** Baseline characteristics of the study population.

Variable	Group A	Group B	Group E	*p*
Gender (Male/female)	10/3	10/3	9/3	1.000
Age (years)	42 (40–47)	43 (40–49)	43.5 (39–48)	0.868
HBsAg (IU/mL)	139.4 (41.8–340.4)	124.6 (85.6–545.5)	5790.2 (1910–8928)	< 0.001 ^∗^
HBV DNA (IU/mL)	< 100	< 100	< 100	NA
ALT (U/L)	27.7 ± 18.8	28.4 ± 7.9	27.9 ± 19.0	0.992
AST (U/L)	23.3 (19.0–28.8)	23.0 (20.2–30.2)	24.1 (19.6–27.9)	0.825
ALP (U/L)	75.0 (65.8–82.5)	78.0 (70.5–83.8)	74.4 (69.1–77.8)	0.600
GGT (U/L)	22.6 (13.7–26.9)	19.1 (13.9–28.3)	15.8 (13.6–17.1)	0.054
ALB (g/L)	46.3 ± 2.5	46.0 ± 3.1	45.1 ± 3.3	0.491
TBil (*μ*mol/L)	16.3 (14.2–24.3)	13.4 (12.3–15.1)	15.3 (12.2–17.1)	0.137
Cr (*μ*mol/L)	66 (62–70)	69 (66–71)	64.5 (59–69)	0.046^a^
BUN (mmol/L)	5.1 (4.3–5.8)	5.5 (4.7–6.3)	5.4 (3.9–6.2)	0.546
WBC (×10^9^/L)	5.68 ± 1.02	5.62 ± 1.29	5.42 ± 1.38	0.860
PLT (×10^9^/L)	200 (185–235)	185 (166–263)	217 (198–231)	0.361
HGB (g/L)	158 (144–165)	147 (142–160)	152 (144–165)	0.200

Abbreviations: ALB, albumin; ALP, alkaline phosphatase; ALT, alanine transaminase; AST, aspartate transaminase; BUN, blood urea nitrogen; Cr, creatinine; GGT, *γ*‐glutamyl transferase; HBsAg, hepatitis B surface antigen; HBV DNA, hepatitis B virus deoxyribonucleic acid; HGB, hemoglobin; NA, not applicable; PLT, platelet; TBiL, total bilirubin; WBC, white blood cell.

^a^Although the median Cr level in Group B was higher than that in other groups, it did not reach statistical significance after multiple comparison correction (corrected *p* > 0.05).

∗The HBsAg level in Group E was significantly higher than that in Group A (*p* < 0.001) and Group B (*p* < 0.001); there was no significant difference between Group A and Group B (*p* = 0.968).

### 3.2. Adjustment for Baseline HBsAg Confounding

Baseline HBsAg was significantly higher in the E group (*p* < 0.001), supporting its role as a potential confounder. After ANCOVA adjustment, 7 of the 9 initially identified differential metabolites retained significance (FDR < 0.05), demonstrating independence from baseline HBsAg; the remaining two showed attenuated significance (FDR near 0.05). The confounder‐corrected OPLS‐DA model maintained robust discrimination (R^2^Y = 0.927, Q^2^ = 0.482; permutation *p* < 0.001), with only mild reduction from the unadjusted model (R^2^Y = 0.982, Q^2^ = 0.560), confirming that metabolic segregation is driven primarily by group allocation rather than baseline HBsAg. Comprehensive data are summarized in Table S2.

### 3.3. Identification of Differential Metabolites

**Figure 1:**
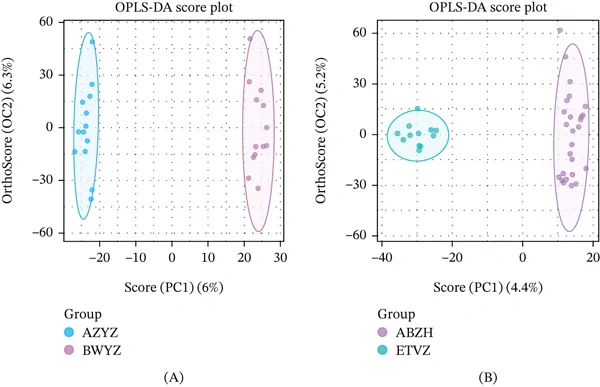
A: OPLS‐DA score plot of Group A versus B; B: OPLS‐DA score plot of Group E versus Z. Note: The abscissa PC1 represents the score value of the first principal component, and the ordinate OC2 represents the score value of the first orthogonal component. Points indicate experimental samples, and colors represent different groups. Group differences are observed from the abscissa, while intragroup differences are observed from the ordinate. The more clustered the intragroup samples and the more scattered the inter‐group samples, the more reliable the results.

Untargeted metabolomic analysis identified 388 serum metabolites, with OPLS‐DA modeling demonstrating significant metabolic separation between Group A and Group B (R^2^Y = 0.995, Q^2^ = 0.545) as well as between Group E and Group Z (R^2^Y = 0.982, Q^2^ = 0.560) (Figures 1A,B). To rule out overfitting derived from the relatively small cohort of 38 CHB patients, 200‐times permutation testing was executed for both OPLS‐DA models. For the Group A versus Group B model, permutation Q2 intercept = 0.04; for Group E versus Group Z model, permutation Q2 intercept = 0.00. Both intercept values were below the critical threshold of 0.05, which confirms no significant overfitting of the multivariate classification models. All QC metrics and permutation test outcomes are summarized in Table S1 and visualized in Figures S3A,B.

**Figure 2:**
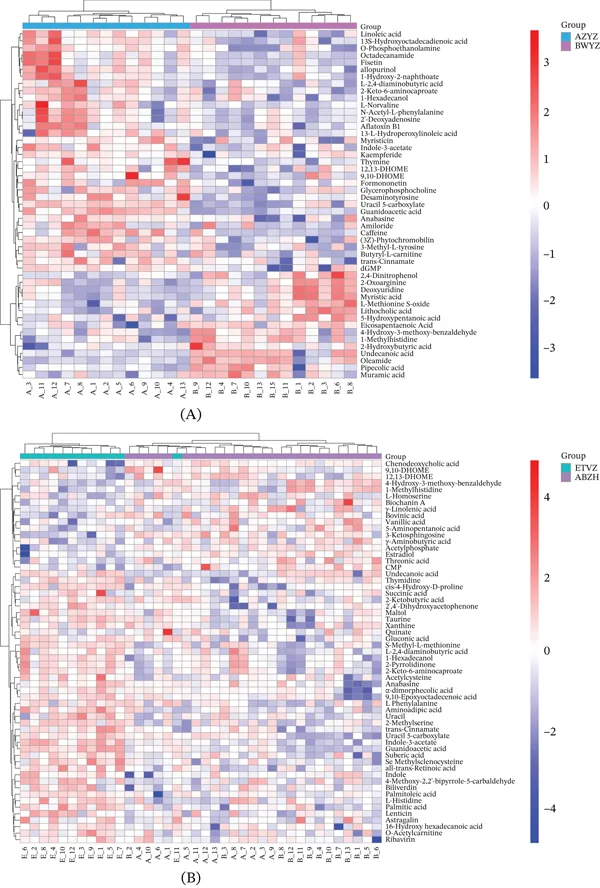
A: Heatmap of differential metabolites between Group A versus B; B: Heatmap of differential metabolites between Group E versus Z. Note: Columns represent samples and rows represent metabolites. The clustering tree on the left is the clustering tree of differential metabolites, and the one at the top is the clustering tree of samples. The gradient color indicates the magnitude of quantitative values: the redder the color, the higher the expression level; the bluer the color, the lower the expression level.

**Figure 3:**
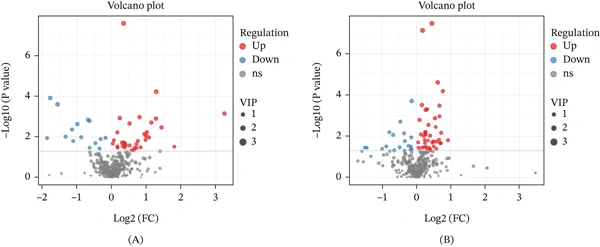
A: Volcano plot of Group A versus B; B: Volcano plot of Group E versus Z. Note: The abscissa represents the log₂(FC) value of metabolite fold change (the larger the absolute value, the more significant the difference), and the ordinate represents the ‐log₁₀ value of *p*‐value (the larger the value, the stronger the statistical significance). Each scatter represents a metabolite, where red dots indicate significantly upregulated metabolites, blue dots indicate significantly downregulated metabolites, and gray dots indicate metabolites that do not meet the difference threshold. The size of the scatter is positively correlated with the VIP value (the larger the size, the higher the importance of the metabolite).

Initial screening (*p* < 0.05 with VIP > 1.0) identified 49 and 59 differential metabolites in Group A versus B and Group E versus Z comparisons, respectively, visualized through heatmaps (Figures 2A,B) and volcano plots (Figure 3A,B) To control the false positive rate and obtain more reliable differential metabolites, we further adopted FDR< 0.05 combined with VIP> 1.0 as the criteria for identifying intergroup differential metabolites. Finally, four differential metabolites were identified between Group A versus B, including two upregulated and two downregulated metabolites. KEGG pathway enrichment analysis revealed that these differential metabolites were mainly enriched in glycine/serine/threonine metabolism, arginine/proline metabolism, and cysteine/methionine metabolism pathways. A total of nine significant differential metabolites were identified between Group E versus Z, among which eight were upregulated and one was downregulated. KEGG analysis indicated that these metabolites were mainly involved in three types of pathways: amino acid metabolism (glycine/serine/threonine metabolism, arginine/proline metabolism, and tryptophan metabolism), immune regulation (retinol metabolism, intestinal immune network for immunoglobulin A (IgA), and selenocompound metabolism), and fatty acid metabolism (fatty acid degradation and synthesis pathways). Detailed differential metabolites and related enriched pathways are shown in Table 2.

**Table 2:** Differential metabolites and related enriched pathways in Group A versus B and Group E versus Z.

Group	Metabolite	Log₂(FC)	*p*	FDR	VIP	KEGG pathway
A vs. B	Guanidinoacetic acid	0.35	2.56E−08	8.44E−05	3.56	Glycine, serine and threonine metabolism/arginine and proline metabolism
	Uracil 5‐carboxylate	1.29	5.94E−05	0.01569	3.08	—
	L‐Methionine S‐oxide	−1.76	0.000122	0.022697	2.78	Cysteine and methionine metabolism
	Oleamide	−1.55	0.000249	0.03383	2.89	—
E vs. Z	Guanidinoacetic acid	0.45	3.37E−08	1.49E−05	3.676	Glycine, serine and threonine metabolism/arginine and proline metabolism
	Indole‐3‐acetate	0.18	7.39E−08	5.85E−05	3.55	Tryptophan metabolism
	All‐trans‐Retinoic acid	0.62	2.45E−05	0.004751	3.00	Intestinal immune network for IgA production/retinol metabolism
	S‐Methyl‐L‐methionine	0.77	6.51E−05	0.009852	2.39	—
	3‐Ketosphingosine	−0.14	0.000197	0.021016	2.77	—
	Se‐Methylselenocysteine	0.16	0.000308	0.028613	2.61	Selenocompound metabolism
	1‐Hexadecanol	0.67	0.000326	0.028757	2.27	fatty acid degradation
	Palmitoleic acid	0.31	0.000489	0.032518	2.13	—
	Palmitic acid	0.27	0.000528	0.033459	2.57	Fatty acid degradation/fatty acid biosynthesis

### 3.4. Predictive Model Construction

**Figure 4:**
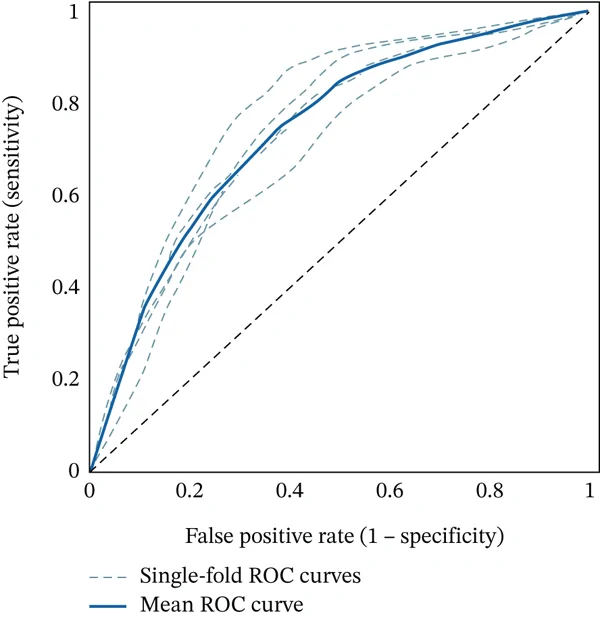
ROC curve of the prediction model for functional cure of CHB.

This study developed a random forest prediction model based on metabolomic data from Group A and Group B. The model incorporated four differential metabolites selected through VIP > 1.0 and FDR < 0.05 criteria as input features. Constructed via a nested 5‐fold cross‐validation approach, the model demonstrated high reliability with an overall classification accuracy of 88.5% (detailed performance metrics in Table 3). ROC curve analysis (Figure 4) further confirmed the model’s excellent discriminative capacity (AUC = 0.920, 95% CI: 0.865–0.975). Feature importance analysis identified L‐Methionine S‐oxide and Oleamide as key metabolic biomarkers distinguishing functionally cured patients (Table 4). Although these results are promising, it should be noted that the model was validated using nested 5‐fold cross‐validation within a single‐center cohort and should be interpreted as exploratory. Independent external validation in larger multicenter cohorts is necessary before clinical application.

**Table 3:** Performance indicators related to the prediction model constructed by the random forest algorithm.

Indicator	Value	95% CI
Accuracy	0.885	0.793–0.977
AUC	0.920	0.865–0.975
Precision	0.882	0.759–1.000
Recall	0.892	0.757–1.000
F1 score	0.886	0.779–0.993

**Table 4:** Ranking and characteristics of metabolites by feature importance generated by the random forest model.

Rank	Metabolite name	KEGG ID	Importance score
1	L‐Methionine S‐oxide	C02989	0.402
2	Oleamide	C19670	0.283
3	Uracil 5‐carboxylate	C03030	0.198
4	Guanidinoacetic acid	C00581	0.117

## 4. Discussion

In this exploratory study, untargeted metabolomics was utilized to characterize metabolic alterations associated with functional cure of CHB achieved through ETV and PEG‐IFN*α* combination therapy. We identified key differential metabolites and pathways associated with functional cure and developed a predictive model based on metabolic biomarkers, which may offer insights for guiding personalized clinical treatment strategies.

We identified four serum metabolites that exhibited significant alterations in the functional cure group. Guanidinoacetic acid and uracil 5‐carboxylate were significantly upregulated, whereas L‐Methionine S‐oxide and oleamide were markedly downregulated in cured patients. These metabolic changes highlight the critical involvement of specific pathways in CHB functional cure.

Guanidinoacetic acid, as the direct precursor of creatine synthesis, is mainly produced in the kidneys and liver through the catalysis of glycine and arginine by glycine amidinotransferase (GATM), and then methylated in the liver by guanidinoacetate methyltransferase (GAMT) to form creatine [22]. The creatine/phosphocreatine system serves as the core for cellular energy buffering and transport [23]. In the field of immunometabolism, arginine and its metabolites (including guanidinoacetic acid and creatine) play pivotal roles in regulating T‐cell receptor (TCR) signaling, proliferation, and effector functions (e.g., IFN‐*γ* secretion) [24]. The upregulation of guanidinoacetic acid may reflect enhanced creatine synthesis via the arginine metabolic pathway in cured patients, which has been reported to support immune cell bioenergetics in other contexts [24]. Although speculative, this metabolic shift might contribute to a microenvironment conducive to antiviral immunity, promoting HBV clearance and HBsAg seroconversion. These findings align with the KEGG pathway analysis, which showed enrichment of guanidinoacetic acid in glycine/serine/threonine metabolism and arginine/proline metabolism pathways.

L‐Methionine S‐oxide is an oxidative product of methionine, a sulfur‐containing essential amino acid involved in protein synthesis, formation of the methyl donor S‐adenosylmethionine, and polyamine synthesis. The production of L‐Methionine S‐oxide is typically associated with elevated reactive oxygen species (ROS) levels and serves as a biomarker of oxidative stress [25, 26]. Its downregulation correlates with the functional cure status and is indicative of reduced oxidative stress in these patients. Given that chronic HBV infection induces oxidative stress [27, 28], the observed metabolic shift may reflect, to some extent, improved redox homeostasis, but the direct contribution of PEG‐IFN*α* requires further validation. These findings align with the involvement of L‐Methionine S‐oxide in the cysteine/methionine metabolic pathway, indicating improved redox homeostasis during the functional cure process.

Oleamide, an oleic acid‐derived fatty acid amide belonging to the endocannabinoid‐like molecular family, exhibits sedative, hypnotic, and immunomodulatory effects [29, 30]. Studies have demonstrated that certain endocannabinoids can suppress immune cell activation (e.g., macrophages, T cells) and inflammatory cytokine production [31]. The downregulation of oleamide aligns with the notion of attenuated immunosuppressive signaling in cured patients, potentially facilitating a more robust antiviral immune response; however, this link remains to be experimentally confirmed. Uracil 5‐carboxylate is one of the intermediate products of uracil nucleotide catabolism. Alterations in uracil metabolism may reflect changes in the balance between nucleotide synthesis and degradation, or be related to the activity of the pyrimidine salvage pathway. Its specific biological significance in CHB functional cure requires further investigation.

Integrating our metabolomic findings with existing literature, we put forward a tentative model of metabolism‐immunity interplay. In this model, ETV‐mediated viral suppression would create a low‐antigen environment, while PEG‐IFN*α* appears to be associated with metabolic shifts that could theoretically optimize immune function—such as enhanced energy metabolism via the arginine/creatine pathway, reduced oxidative stress indicated by decreased L‐methionine S‐oxide, and attenuated immunosuppressive signaling reflected by lowered oleamide levels. If validated, such putative metabolic‐immune crosstalk might explain the synergistic effects observed clinically.

When comparing the combination therapy group as a whole with the ETV monotherapy group, we found more extensive metabolic differences involving pathways such as amino acid metabolism, immune regulation, and fatty acid metabolism. This clearly indicates that the addition of PEG‐IFN*α* significantly alters the systemic metabolic state of CHB patients, far beyond the viral suppression achieved by ETV monotherapy alone. For instance, the tryptophan metabolite indole‐3‐acetate is significantly increased in the combination group. This metabolite is associated with gut microbiota metabolism and can affect immunity through the aryl hydrocarbon receptor pathway [32], suggesting that the gut‐liver axis may play a role in the response to combination therapy. The enrichment of the retinol metabolism pathway also suggests that the regulation of immune cell (such as dendritic cells and B cells) differentiation and function may be one of the important mechanisms of the combination therapy. These findings further indicate that the combination therapy exerts a synergistic antiviral effect through multipathway and multilevel immunometabolic regulation.

In addition, the random forest prediction model we constructed showed excellent performance in distinguishing patients in the cured group from those in the noncured group. The model highlighted L‐methionine S‐oxide and oleamide as key markers for differentiating the functional cure group. This provides a new approach for the early clinical identification of patients who may benefit from ETV‐PEG‐IFN*α* combination therapy and achieve functional cure.

Several limitations of this study should be acknowledged. First, the retrospective design and relatively small sample size (*n* = 38) limit the statistical power and generalizability of the findings. Second, because of nonrandomized grouping based on real‐world clinical indications, significant between‐group differences in baseline HBsAg levels were present. Although the core metabolic differences remained statistically significant after adjustment for baseline HBsAg via ANCOVA, residual‐based OPLS‐DA, and partial correlation analyses, residual confounding cannot be entirely excluded. Third, several clinically relevant variables that may influence therapeutic response—including fibrosis stage, liver stiffness measured by transient elastography, duration of HBV infection, HBV genotype, detailed history of prior antiviral therapy, and quantitative anti‐HBs titers—were not available or systematically collected. Although the exclusion criteria minimized the inclusion of patients with advanced liver disease, unmeasured fibrosis severity may still introduce residual confounding. The absence of HBV genotype data is a notable limitation, given its potential impact on interferon responsiveness; however, the geographically and ethnically homogeneous nature of our cohort (Shandong, China, where genotypes B and C predominate) may partially mitigate this concern. Fourth, the random forest predictive model was developed and validated using nested five‐fold cross‐validation within a single‐center retrospective cohort, and independent external validation in separate patient populations is still lacking. Therefore, the predictive performance of the model should be interpreted as exploratory rather than clinically definitive. Fifth, although metabolomics provides a snapshot of biochemical alterations, this study does not provide direct functional evidence of the proposed mechanisms; the observed metabolic changes represent associations rather than proof of causality. Future prospective studies with larger cohorts, propensity score matching or baseline HBsAg‐matched designs, comprehensive clinical phenotyping (including elastography, HBV genotyping, and serial quantitative serological markers), longitudinal metabolomic sampling, and multiomics integration are warranted to validate and extend these findings.

## 5. Conclusions

This study characterizes metabolic alterations associated with functional cure achieved by ETV‐PEG‐IFN*α*‐2b treatment, with core changes involving energy metabolism, redox balance, and immune regulation. The proposed metabolism‐immunity synergy model suggests that PEG‐IFN*α*‐associated metabolic shifts may contribute to enhanced immune function on the basis of viral suppression by ETV. The identified key metabolites demonstrate potential as predictive biomarkers, pending validation in larger prospective cohorts.

## Author Contributions

Junling Wang: investigation, data curation, formal analysis, visualization, validation, writing—original draft. Xianghong Kong: investigation, resources, validation, writing—review and editing. Huiping Xu: investigation, resources, validation, writing—review and editing. Wei Zhang: methodology, software, data curation, validation, resources, writing—review and editing. Peng Peng: conceptualization, methodology, supervision, project administration, formal analysis, resources, writing—review and editing.

## Funding

No funding was received for this manuscript.

## Conflicts of Interest

The authors declare no conflicts of interest.

## Supporting Information

Additional supporting information can be found online in the Supporting Information section.

## Supporting information


**Supporting Information 1** Detailed conditions for ultra‐performance liquid chromatography/mass spectrometry (UPLC‐MS) analysis of samples.

**Supporting Information 2** Figure S1: QC Sample PCA Score Plots. The tight clustering of QC samples in the PCA score plots demonstrates excellent analytical reproducibility and stability of the LC‐MS system throughout the entire analysis run, confirming the reliability of the acquired metabolomic data.

**Supporting Information 3** Figure S2: Relative Standard Deviation (RSD) Distribution Plots. These histograms show the distribution of relative standard deviations (RSD%) of metabolite intensities across all QC samples. The majority of metabolites showed RSD values below 30%, which is well within the acceptable range for untargeted metabolomics studies, further confirming the high quality and reliability of our data.

**Supporting Information 4** Figure S3: Permutation test plots of two OPLS‐DA models. (A) Permutation plot for Group A versus Group B; (B) Permutation plot for Group E versus Group Z. The horizontal dashed line represents Q^2^ = 0.05 threshold; all random permutation Q^2^ values and model intercepts are below this cutoff, indicating absence of overfitting.

**Supporting Information 5** Table S1: QC performance metrics and permutation test results for OPLS‐DA models. Table S2: Differential metabolites before and after adjustment for baseline HBsAg using ANCOVA.

## Data Availability

The data that support the findings of this study are available from the corresponding author upon reasonable request.
