# Supplementary material for: Metabolomics Reveals Metabolic Characteristics of Functional Cure in Chronic Hepatitis B Treated With Entecavir Combined With Pegylated Interferon Alpha
Source: Biomed Res Int. 2026 Sep 3;2026:1031453. doi: 10.1155/bmri/1031453 (PMC13542708; doi:10.1155/bmri/1031453)
Supplement: Supplementary file 1 [file BMRI-2026-1031453-s001.docx]

**Detailed conditions for ultra-performance liquid chromatography/mass spectrometry (UPLC-MS) analysis of samples**

*Liquid chromatography conditions*

The LC analysis was performed on a Vanquish UHPLC System (Thermo Fisher Scientific, USA). Chromatography was carried out with an ACQUITY UPLC ® HSS T3 (2.1×100 mm, 1.8 µm) (Waters, Milford, MA, USA). The column maintained at 40 ℃. The flow rate and injection volume were set at 0.3 mL/min and 2 μL, respectively. For LC-ESI (+)-MS analysis, the mobile phases consisted of (B2) 0.1% formic acid in acetonitrile (v/v) and (A2) 0.1% formic acid in water (v/v). Separation was conducted under the following gradient: 0~1 min，8% B2；1~8 min，8%~98% B2；8~10 min，98% B2；10~10.1 min，98%~8% B2；10.1~12 min，8% B2. For LC-ESI (-)-MS analysis, the analytes was carried out with (B3) acetonitrile and (A3) ammonium formate (5mM). Separation was conducted under the following gradient: 0~1 min，8% B3；1~8 min，8%~98% B3；8~10 min，98% B3；10~10.1 min，98%~8% B3；10.1~12 min，8% B3 [1].

*Mass spectrum conditions*

Mass spectrometric detection of metabolites was performed on Orbitrap Exploris 120 (Thermo Fisher Scientific, USA) with ESI ion source. Simultaneous MS1 and MS/MS (Full MS-ddMS2 mode, data-dependent MS/MS) acquisition was used. The parameters were as follows: sheath gas pressure, 40 arb; aux gas flow, 10 arb; spray voltage, 3.50 kV and -2.50 kV for ESI(+) and ESI(-), respectively; capillary temperature, 325 ℃; MS1 range, m/z 100-1000; MS1 resolving power, 60000 FWHM; number of data dependant scans per cycle, 4; MS/MS resolving power, 15000FWHM; normalized collision energy, 30%; dynamic exclusion time, automatic [2].

REFERENCES

1. Zelena E, Dunn WB, Broadhurst D, et al. Development of a Robust and Repeatable UPLC-MS Method for the Long-Term Metabolomic Study of Human Serum. Anal Chem. 2009;81(4):1357-64. https://doi.org/10.1021/ac8019366.

2.Want EJ, Masson P, Michopoulos F, et al. Global metabolic profiling of animal and human tissues via UPLC-MS. Nat Protoc. 2013;8(1):17-32. https://doi.org/10.1038/nprot.2012.135.
