# Supplementary material for: Metabolomics Reveals Metabolic Characteristics of Functional Cure in Chronic Hepatitis B Treated With Entecavir Combined With Pegylated Interferon Alpha
Source: Biomed Res Int. 2026 Sep 3;2026:1031453. doi: 10.1155/bmri/1031453 (PMC13542708; doi:10.1155/bmri/1031453)
Supplement: Supplementary file 2 [file BMRI-2026-1031453-s005.docx]

**Supplementary Table 1.** QC performance metrics and permutation test results for OPLS-DA models

| Metric | A vs. B | E vs. Z |
| --- | --- | --- |
| R^2^X (cumulative) | 0.122 | 0.0981 |
| R^2^Y (cumulative) | 0.996 | 0.982 |
| Q^2^ (cumulative) | 0.511 | 0.560 |
| Permutation R^2^ intercept | 0.990 | 0.980 |
| Permutation Q^2^ intercept | 0.040 | 0.000 |

**Supplementary Table 2.** Differential metabolites before and after adjustment for baseline HBsAg using ANCOVA

| Metabolite | Original VIP | Original FDR | Adjusted ANCOVA P | Adjusted FDR | Partial r (adjusted for HBsAg) |
| --- | --- | --- | --- | --- | --- |
| Guanidinoacetic acid | 3.676 | 1.49E-05 | < 0.001 | 2.31E-04 | 0.724 |
| Indole-3-acetate | 3.55 | 5.85E-05 | < 0.001 | 4.72E-04 | 0.698 |
| All-trans-Retinoic acid | 3.00 | 0.004751 | 0.0012 | 0.0078 | 0.587 |
| S-Methyl-L-methionine | 2.39 | 0.009852 | 0.0031 | 0.0127 | 0.542 |
| 3-Ketosphingosine | 2.77 | 0.021016 | 0.0076 | 0.0245 | 0.496 |
| Se-Methylselenocysteine | 2.61 | 0.028613 | 0.0112 | 0.0318 | 0.463 |
| 1-Hexadecanol | 2.27 | 0.028757 | 0.0124 | 0.0325 | 0.455 |
| Palmitic acid | 2.57 | 0.033459 | 0.0198 | 0.0487 | 0.412 |
| Palmitoleic acid | 2.13 | 0.032518 | 0.0217 | 0.0512 | 0.398 |
